# The influence of sensation seeking on college student sports lottery purchasing behavior: a moderated mediation model

**DOI:** 10.3389/fpsyg.2025.1537328

**Published:** 2025-02-19

**Authors:** Kanglin Wang, Fen Qiu

**Affiliations:** School of Physical Education, Wuhan University of Technology, Wuhan, China

**Keywords:** sensation seeking, college students, sports lottery purchasing behavior, fear of missing out, perceived risk

## Abstract

**Objective:**

The study aims to explore how sensation seeking relates to college students sports lottery purchasing behavior, mediated by fear of missing out and moderated by perceived risk.

**Methods:**

A study surveyed 409 college students using various scales to assess sensation seeking, sports lottery purchasing behavior, fear of missing out, and perceived risk. Data analysis included descriptive stats, t-tests, partial correlations, mediation and moderation effects, and Bootstrap testing to understand the psychological drivers behind sports lottery purchases among students.

**Results:**

(1) Sensation seeking significantly and positively predicted college students sports lottery purchasing behavior; (2) Fear of missing out played a partial mediating effect in the influence of sensation seeking on college students sports lottery purchasing behavior; (3) Perceived risk had a moderating effect in the second half of the path of the mediating effect of fear of missing out.

**Conclusion:**

The study concludes that sensation seeking directly affects college students sports lottery purchasing behavior, mediated by fear of missing out. The mediating effect is moderated by perceived risk, highlighting the complexity of psychological factors in risk-taking behaviors. This insight calls for tailored interventions addressing these multifaceted factors.

## 1 Introduction

Sports lottery purchasing behavior is the specific actions and internal activities displayed by individuals through the purchase of lottery tickets to satisfy their own needs (Na, 2021). This is not only limited to the actual purchase behavior but also covers the psychological considerations before the purchase decision, as well as the prize redemption process and the ensuing psychological fluctuations and changes experienced by the individual after the winning results are revealed. According to the frequency of purchase, purchase amount, and pre-purchase studies, it can be divided into rational and irrational lottery purchases (Chen et al., 2014). The “14th Five-Year Plan” sports lottery development plan puts forward that the high-quality development of sports lottery needs to focus on the three centers of “risk, brand and channel” to coordinate all the work and promote the correct development of the sports lottery industry (Sport, 2024a,b,c). China has been committed to giving full play to the economic role of the sports lottery industry in recent years to promote the high-quality development of China’s sports industry. China’s sports lottery has the characteristics of national issuance, public welfare, credibility, sustainability entertainment. These attributes make the sports lottery play an important role in raising funds for sports and promoting the development of sports, according to the statistics, by 2024, China sports lottery national unified issuance of 30 years, China sports lottery has raised a total of more than 810 billion yuan of public welfare (Sport, 2024a,b,c), and it plays a pivotal role in driving national development and social welfare undertakings. It effectively allocates resources, significantly contributing to infrastructure building, public service expansion, and overall social progress. Under the backdrop of rapid economic development, college students tend to show a heightened willingness to embrace and engage in popular and recreational activities, signifying a robust potential for future consumption. Sports lottery, as an entertaining and exciting way of consumption, will attract the attention of some college students. College students are at a crucial stage of personal development, with high adaptability and a strong inclination toward new trends. They are also active participants in the digital age, often exposed to various marketing and promotional activities related to the sports lottery through social media and online platforms. In 2023, China Sports Lottery’s sales reached 385.255 billion yuan and in the first 11 months of 2024, the sales volume of China Sports Lottery reached 377.091 billion yuan, with a year-on-year growth of 7.9% (Sport, 2024a,b,c), and the number of lottery players has shown a tendency to become younger in recent years, with college students and young people accounting for 36.4% of the total amount of purchases in 2023. As the main force of social consumption in the future, the cultivation of college students’ consumption habits is an important consideration for the layout of China Sports Lottery in the future. The money concepts and spending habits of college students are not yet mature, and they are vulnerable to internal personality and external stimuli for consumption. The news of “college students borrowing campus loans to purchase lottery tickets” and “college students becoming addicted to purchasing lottery tickets” are also emerging one after another. According to existing research, college students’ motives for purchasing sports lotteries are diverse, and some of them are often benign (Ariyabuddhiphongs, 2011), so how to guide college students to purchase sports lotteries in moderation and maintain a correct mindset of purchasing sports lotteries has become a problem that needs to be solved urgently.

## 2 Literature review and hypotheses

### 2.1 Sensation seeking and sports lottery purchasing behavior

According to Eysenck’s theory of personality dimensions and implicit personality theory, it has been found that personality traits affect consumers’ consumption cognition, which in turn affects people’s consumption decisions and consumption behavior (Ko et al., 2017). The concept of Sensation Seeking was originally derived from sensory deprivation experiments (Zuckerman, 1990), which was defined as a personality trait by Zuckerman in 1979, and through continuous research, it was defined as the search for variable, novel, complex, and intense sensations and experiences, and the achievement of these experiences by taking physical, social, legal and economic risks (Zuckerman, 1996). It can be seen that sensation seeking is significantly characterized by the individual’s quest for novelty, change, or stimulation, which requires not only the performance of certain actions but also the assumption of certain risks to obtain a stimulating experience (Zuckerman, 2014). According to existing studies, sensation seeking affects people’s consumption habits (Kiss et al., 2024), impulse spending (Farhat et al., 2012), and the choice of high-risk sports such as rock climbing (Martha et al., 2009); and, high perceived winds show a significant positive correlation to betting on sports (Sanmartín et al., 2023). Therefore, this study proposes Hypothesis 1: Sensation seeking positively predicts the sports lottery purchasing behavior of college students.

### 2.2 Mediation effect of fear of missing out

Fear of Missing Out (FoMO) is a diffuse state of anxiety in individuals arising from the fear of missing out on novel experiences or positive events that others have had, a pervasive concern that others may have beneficial experiences that they have not experienced (Przybylski et al., 2013), characterized by a desire to constantly be in tune with one’s surroundings. Wegmann again made a more nuanced distinction between fear of missing out in 2017, where they proposed that an individual’s innate tendency to have a fear of missing out is the trait variable aspect of fear of missing out, whereas an individual’s experiencing different levels of fear of missing out in different situations is the state variable aspect (Wegmann et al., 2017). Przybylski noted that there is a relationship between personality traits and fear of missing out, with one study noting that high sensation-seeking populations in the same environment are more prone to fear of missing out (Meng et al., 2023). In recent years, the fear of missing out has been increasingly applied to consumer behavior research and marketing strategy elements, in the study of impulsive consumption behavior and conspicuous consumption, the fear of missing out will induce people’s consumption motivation through the change of emotions (Dursun et al., 2023), “hunger marketing,” “Netflix economy “and other marketing means are based on people’s emotional changes to promote consumption, and in the investigation of college students’ motives for purchasing lottery tickets and the mechanism of influencing factors, impulse consumption, following the trend of consumption and love of sports and desire for prizes are the main motives for college students to purchase sports lottery tickets (Browne et al., 2023). Therefore, this study proposes hypothesis 2: the fear of missing out plays a mediating effect in the influence of sensation seeking on college students’ sports lottery purchasing behavior.

### 2.3 Moderate effect of perceived risk

Perceived risk is defined as the risk that consumers take after making a purchase of a product or service based on the uncertainty of the reward the diversity of the purpose of the purchase, and the subjective expectation of uncontrollable outcomes that consumers take when making a shopping decision (Cox, 1967). Cunningham proposed in 1964 that consumers pay for a purchase that may not satisfy their goals before making the payment. Before making a purchase, consumers perceive risk by creating the possibility that the purchase may not satisfy their goals, which may include social consequences, loss of money, loss of time, etc. (Cunningham, 1967). Sports lotteries, like blind boxes, have unpredictable purchase outcomes, and when researchers studied repurchase intentions for blind boxes, perceived risk moderates people’s perceived value of the product and increases customers’ repurchase intentions (Shapiro et al., 2019). According to the ABC theory proposed by the psychologist Ellis, it is believed that customers will be affected by the negative emotions (Antecedent) caused by some triggering events before generating a purchase action (Consequence), and will also be influenced by the individual’s cognitive beliefs (Belief) generated by the cognition and evaluation of the triggering event A (Wu et al., 2020). Perceived risk can both inhibit college students’ impulse to purchase lottery tickets due to the fear of missing out, and make them weigh the risks and benefits more rationally in their decision-making. It is found that consumers’ perceived value and risk perception of a product will affect their willingness to purchase the product, especially in experiential consumption and leasing activities (Wagner et al., 2020), and regulate consumers’ purchase motivation and emotional fluctuations. Therefore, this study proposes Hypothesis 3: Perceived risk may moderate the relationship between fear of missing out and college students’ sports lottery purchasing behavior.

**Figure 1:**
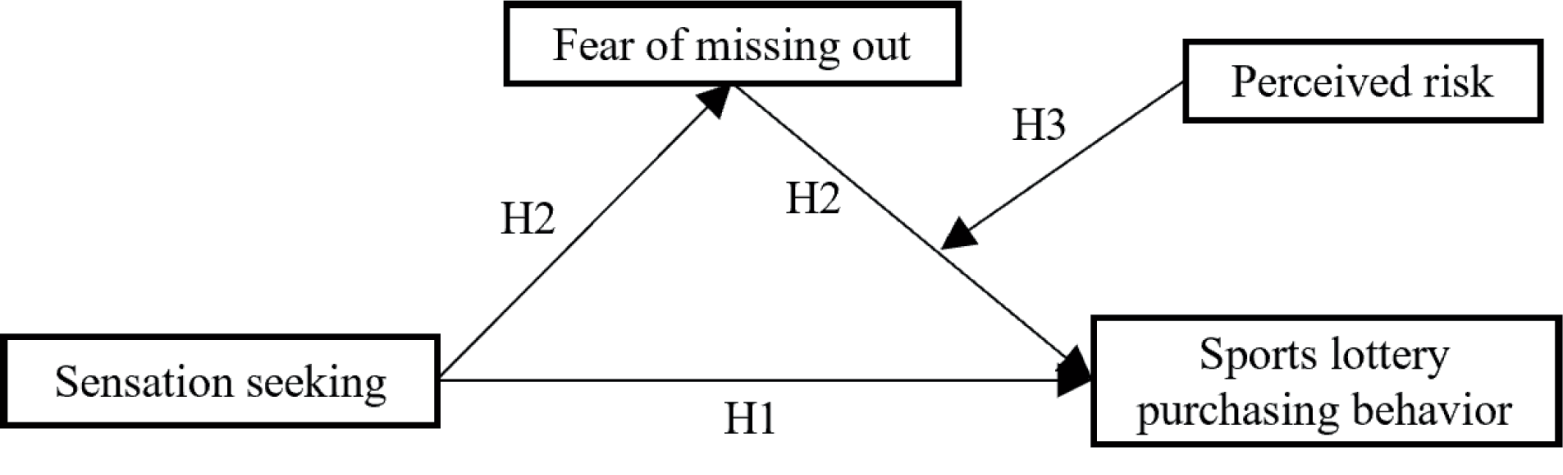
A hypothetical model with moderated mediating effects.

In summary, sensation seeking positively predicts college students’ sports lottery purchasing behavior. However, in the context of deepening learning theory, little literature has explored the relationship between sensation seeking and sports lottery purchasing behavior from the perspective of mood change, especially after introducing fear of missing out as a mediating variable and perceived risk as a moderating variable. Therefore, this study proposes research hypotheses and constructs a theoretical hypothesis model based on theoretical analysis (see Figure 1).

## 3 Materials and methods

### 3.1 Research subjects

In this study, convenience sampling was used to select Hubei Province, Hebei Province, and Beijing Municipality. Simple random sampling was used to select college students enrolled in five universities, as the target respondents, and the sampling unit was the class; a total of 450 questionnaires were collected, and after 45 invalid questionnaires were excluded, there were 409 valid questionnaires (90.89%). The sample included 255 male participants (62.3%) and 154 female participants (37.7%). The mean age was 22.21 years (SD = 3.927). Among them, 245 were undergraduates (59.9%) and 164 were graduate students (40.1%). For mainstream lottery types, 60.7% of participants preferred instant lotteries, followed by quiz lotteries (20.4%) and draw-based lotteries (18.8%).

### 3.2 Measurement tools

#### 3.2.1 Sensation seeking

The scale was developed by Steinberg et al. (2008) and was revised by Li et al. (2010). It is a unidimensional scale with a total of 6 question items, and is scored on a 5-point Likert scale (1 = “not at all conforming,” 5 = “fully conforming”), with the total score as the “total score,” and the total score is used as the final score, with higher scores indicating higher levels of sensation seeking. The Cronbach *α* coefficient in this study is 0.867.

#### 3.2.2 Fear of missing out

The scale was developed by Przybylski et al. (2013) and was translated by Xie et al. (2018), which is a one-dimensional scale with 10 question items, scored on a 5-point Likert scale (1 = “not at all conforming,” 5 = “fully conforming “), and the total score is used as the final score, with higher scores indicating higher levels of fear of missing out. The Cronbach α in this study is 0.855.

#### 3.2.3 Sports lottery purchasing behavior

The scale was developed by Xu et al. (2023), which consists of 15 questions, including five dimensions: chasing behavior, control disorder, purchasing behavior on behalf of others, superstitious behavior, and number association, and is scored on a 5-point Likert scale (1 = “not at all consistent,” 5 = “Fully Compliant”). Based on the questions in the original scale, the directionality of the questions was strengthened by modifying the core word “lottery” in the original scale to “sports lottery.” The revised scale was tested with *χ*^2^/df = 2.330, RESEA = 0.057, GFI = 0.945, AGFI = 0.914, CFI = 0.945, IFI = 0.945, and TLI = 0.927 in the validated factor analysis (CFA), and was found to be in line with the needs of this study, the Cronbach alpha coefficient in this study was 0.859.

#### 3.2.4 Perceived risk

The scale compiled by Cui (2019) was used for the measurement, with six dimensions and 17 question items, namely perceived product risk, perceived financial risk, perceived after-sales service risk, perceived psychological risk, and perceived social risk. Based on the special attributes of sports lottery in this study, the dimension of perceived after-sales service risk (3 items) was deleted, and the core word “product” was modified to “sports lottery,” which resulted in 5 dimensions, 14 items, and a Likert scale of 5 (1 = “not at all”). “not at all,” (5 = “fully compliant”). The revised scale was tested with *χ*^2^/df = 2.041, RESEA = 0.051, GFI = 0.954, AGFI = 0.932, CFI = 0.909, IFI = 0.933 in the validated factor analysis (CFA). The scale was tested to meet the investigative needs of the present study, with a Cronbach alpha coefficient was 0.867.

### 3.3 Data collection and analysis

In this study, we visited multiple universities to conduct surveys. Prior to commencing, we obtained the subjects’ consent and had them sign informed consent forms. The questionnaires distributed did not pertain to any sensitive topics. Subjects were given the opportunity to read the questionnaires independently. Once completed, the questionnaires were promptly retrieved. We employed SPSS 26.0 software to rigorously execute a reliability analysis, a common method bias assessment, descriptive statistical evaluations, and correlation analyses of the study scale. Furthermore, we leveraged AMOS 28.0 to conduct a comprehensive validation factor analysis and verify the scale’s construct validity. To complement these analyses, we adopted the PROCESS plugin, developed by Preacher and Hayes (2004), to conduct sophisticated model testing and simple slope analyses, adhering to the stringent standards expected of academic journal publications (Preacher et al., 2007).

## 4 Results and analysis

### 4.1 Common method bias test

Procedural control and Harman’s one-factor test were used to examine possible common method bias in the survey process. (1) Program control. During the distribution of the questionnaire, it was emphasized in the title of the introduction that “this study is for scientific research only,” and the confidentiality and anonymity of the data were emphasized to minimize the interference of external factors and reduce the error of personal psychology. (2) Harman’s one-way test. Harman’s one-way test was conducted on the collected data, and the results showed that there were 11 factors with eigenvalues greater than 1, and the variance explained rate of the first common factor was 19.4%, which was less than the critical criterion of 40%. It indicates that there is no significant common method bias in this study.

### 4.2 Correlation analysis

The results of descriptive statistics and correlation analysis of each variable are shown in Table 1, which shows that there is a significant positive correlation between sensation seeking and sports lottery purchasing behavior, fear of missing out, and perceived risk; there is a significant positive correlation between sports lottery purchasing behavior and fear of missing out and perceived risk; there is also a significant positive correlation between fear of missing out and perceived risk, which is in line with the conditions of the test of mediating effect and moderating effect.

**Table 1:** Mean, standard deviation, and correlation coefficient of each variable.

Variable	*M*	SD	1	2	3	4
Sensation seeking	2.770	0.836	1			
Sports lottery purchasing behavior	2.746	0.676	0.362^**^	1		
Fear of missing out	2.755	0.714	0.252^**^	0.397^**^	1	
Perceived risk	2.833	0.698	0.275^**^	0.415^**^	0.200^**^	1

*P < 0.05, **p < 0.01, ***p < 0.001, the same below.

### 4.3 Test of the mediating effect of fear of missing out

The mediation model (Model 4) of the PROCESS plug-in designed by Hayes was used to test and verify the mediating effect of fear of missing out between sensation seeking and sports lottery purchase behavior. The results are shown in Table 2, sensation seeking can positively predict sports lottery purchasing behavior (*β* = 0.226, *t* = 6.215), and fear of missing out (*β* = 0.216, *t* = 5.259); when sensation seeking and fear of missing out predicted sports lottery purchasing behavior at the same time, the fear of missing out significantly predicted sports lottery purchasing behavior positively (*β* = 0.310, *t* = 7.264), and at this time, the sensation seeking’s prediction of sports lottery purchasing behavior remained significant (*R*^2^ = 0.231, *F* = 60.964).

**Table 2:** Tests the mediation model of fear of missing.

	Sports lottery purchasing behavior		Fear of missing out	
	*β*	*t*	*β*	*t*
Sensation seeking	0.226	6.215***	0.216	5.259***
Fear of missing out	0.310	7.264***		
*R* ^2^	0.231		0.636	
*F*	60.964		27.661	

For further mediation effect analysis, the relationship between sensation seeking and sports lottery purchasing behavior was calculated using fear of missing out as the mediating variable, sensation seeking as the independent variable, and sports lottery purchasing behavior as the dependent variable, including the direct effect as well as the mediation effect mediated by fear of missing out. As can be seen from Table 3, the direct effect of sensation seeking on sports lottery purchasing behavior is 0.226, with a 95% confidence interval of [0.155, 0.298], and the indirect effect mediated by fear of missing out is 0.067, with a 95% confidence interval of [0.035, 0.103]. Combining the above data shows that the confidence interval for the direct effect does not contain zero, nor does the confidence interval for the indirect effect. This indicates that the indirect effect of sensation seeking on sports lottery purchasing behavior is statistically significant; the direct effect of sensation seeking on sports lottery purchasing behavior remains significant after controlling for the effect of fear of missing out. Therefore, fear of missing out was partially mediated between sensation seeking and sports lottery purchasing behavior and the mediation percentage was 22.87%.

**Table 3:** Analysis of the mediating effect of fear of missing out.

	Effect	BootSE	BootLLCI	BootULCI	Effect size ratio
Indirect effect	0.067	0.021	0.035	0.103	22.87%
Direct effect	0.226	0.037	0.155	0.298	77.13%
Total effect	0.293	0.037	0.219	0.366	100%

### 4.4 The moderating role of perceived risk in mediating effects

Utilizing Model 14 within the PROCESS framework, the Bootstrap method was implemented to scrutinize the intricate relationships among perceived risk in sensation seeking, sports lottery purchasing behavior, and fear of missing out. The results, as shown in Table 4, showed that sensation seeking positively predicted fear of missing out and college students’ sports lottery purchasing behavior, and the interaction term of fear of missing out and perceived risk had a significant predictive effect on college students’ sports lottery purchasing behavior, suggesting that perceived risk moderates the relationship between sensation seeking and college students’ sports lottery purchasing behavior, i.e., it moderates the second half of the path of mediation effect, and the mediation model with moderation was significant (*R*^2^ = 0.3382, *F* = 51.6145, *p* < 0.001), with a 95% confidence interval of [0.102, 0.310].

**Table 4:** Tests of moderated mediating effect.

	Sports lottery purchasing behavior				
	*β*	se	*t*	LLCI	ULCI
Sensation seeking	0.163	0.035	4.6826***	0.095	0.232
Fear of missing out	0.261	0.040	6.4995***	0.182	0.339
Perceived risk	0.275	0.041	6.639***	0.194	0.356
Fear of missing out × Perceived risk	0.206	0.053	3.8953***	0.102	0.310
*R* ^2^	0.3382				
*F*	51.6145				

**Figure 2:**
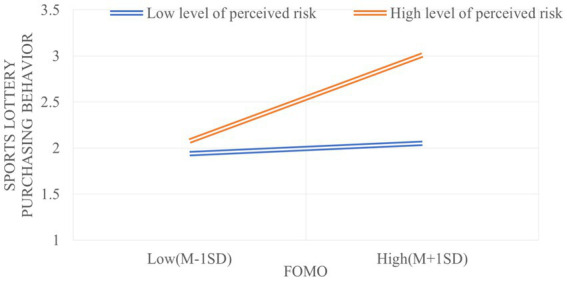
Mediating effects at different levels of perceived risk.

As shown by further simple slope analysis (see Figure 2), the effects of fear of missing out on college students’ sports lottery purchasing behavior were significant under the moderating impact of different levels of perceived risk. For subjects with low perceived risk level (M−1SD), the positive predictive effect of fear of missing out on sports lottery purchasing behavior is significant (*B*_simple_ = 0.025, *t* = 2.0794, *p* < 0.05); for subjects with high perceived risk level (M + 1SD), the positive predictive effect of fear of missing out on sports lottery purchasing behavior is still significant (*B*_simple_ = 0.087, *t* = 7.6469, *p* < 0.001). The moderating interaction increases gradually with the level of perceived risk, and the effect of fear of missing out on college students’ sports lottery purchasing behavior is much higher at high levels of perceived risk than at low levels of perceived risk.

## 5 Discussion

### 5.1 Direct impact

The study’s results confirm the validity of hypothesis 1, that is, sensation seeking positively predicts college students’ sports lottery purchasing behavior. Research has shown that in the college student population, individuals with high sensation seeking tend to be more easily attracted by the uncertainty and potential high returns brought by sports lottery (Brown et al., 2018). Combined with the social cognitive theory, when the social environment or social circle in which college students live has a positive attitude toward purchasing sports lottery tickets, they are more likely to be influenced by this atmosphere and then produce purchasing behavior. Especially for the highly sensation-seeking college student group, they pay more attention to the evaluation and recognition around them, so they are more likely to be influenced by social cognition, thus increasing the possibility of purchasing sports lottery (Tamon et al., 2022). Sports lottery purchase itself is a behavior full of excitement and adventure, and the uncertainty of its outcome provides buyers with a strong emotional value experience. In addition, college students may also be influenced by economic factors, pursuing the potential rewards brought by sports lottery, college students are more unified in the source of funds, and their strong disposability will make it easier for them to purchase lottery tickets (Hedenus, 2011).

### 5.2 Mediating effect of fear of missing out

The results of the study confirm that Hypothesis 2 holds, that is, the fear of missing out plays a mediating effect in the process of sensation seeking positively predicting college students’ sports lottery purchasing behavior. This means that high sensation-seeking individuals are driven by the fear of missing out while pursuing exciting experiences, which in turn increases the likelihood of purchasing sports lottery tickets. On the one hand, the fear of missing out gives individuals a strong urge to purchase because they are worried about missing out on the possible benefits; on the other hand, sensation seeking makes individuals more inclined to pursue this thrilling experience, thus increasing the likelihood of purchase. In addition, information about winning the lottery on social media may exacerbate individuals’ fear of missing out (Clor-Proell et al., 2020). When individuals see reports of others winning the lottery, they may experience a strong sense of anxiety that they have missed the same opportunity (Beckert and Lutter, 2013). This anxiety will further push them to purchase lottery tickets, and college students who frequently browse social media have a higher willingness to purchase sports lottery tickets. Values and norms in the cultural background, on the other hand, may influence an individual’s views and attitudes toward sports lottery tickets, and it has been found that an individual’s psychological traits, such as self-control and impulsivity, also affect their purchase decisions, and individuals with lower self-control are more likely to be driven by impulsivity and immediate desire, which increases the likelihood of purchasing lottery tickets, and individuals with lower self-control are more likely to engage in high-risk activities (Shepperd and Forsyth, 2023) and purchase lottery tickets. This suggests that self-control ability plays an important role in influencing individuals’ lottery purchasing behavior.

### 5.3 Moderating effect of perceived risk

The results of the study also confirm that Hypothesis 3 is valid, and under the framework of the perceived risk theory, this study delves into the influence of perceived risk on sports lottery purchase behavior. Specifically, when college students perceived a higher risk in purchasing sports lottery tickets, they were more likely to develop a fear of missing out on potentially high returns, i.e., the fear of missing out. This strong fear of missing out will further motivate individuals to increase their purchasing behavior in the expectation of avoiding missing out on potentially large gains. When college students are driven by the fear of missing out, they will be more inclined to purchase sports lottery tickets to avoid the regret and anxiety of missing out on potentially high gains. The multidimensional decision-making model emphasizes the important role of emotional factors in the decision-making process in addition to rational analysis. The cognitive and emotional processes of college students’ lottery purchasing have a significant impact on their repurchasing behavior (Zhou and Wu, 2024). Perceived risk, as an important decision-making factor, directly affects college consumers’ willingness to purchase, while sensation-seeking and fear of missing out act as affective factors that further influence individuals’ decision-making process (Shin and Montalto, 2015). The level of perceived risk will affect consumer behavior. College students with higher perceived risk levels are more affected by emotions and personal impulses than college students with lower perceived risk levels.

## 6 Conclusion and suggestions

### 6.1 Conclusion

In summary, this study provides an in-depth analysis of the roles played by the fear of missing out and perceived risk in college students’ sports lottery purchasing behavior, and how they influence and shape this particular consumer behavior. The results show that when college students purchase sports lottery tickets, their internal fear of potentially missing out on a chance to win and the degree of perceived risk are key factors driving their purchasing decisions.

### 6.2 Suggestions

This research not only gives new insights into their lottery purchase behavior but also uncovers the psychological hurdles they face when chasing excitement and potential profits. It thus paves the way for promoting their rational lottery engagement.

In terms of product design, the sports – lottery industry should create products with interactivity and educational value for college students, especially those who seek excitement. For instance, incorporate sports – knowledge quizzes into lottery games, where correct answers can increase winning odds, thus combining entertainment with learning (Du et al., 2019).

Regarding marketing, instead of using aggressive sales methods, the industry should enhance its brand image by sponsoring campus sports events. During these events, introduce lottery products through interactive games rather than direct sales. Meanwhile, utilize social – media platforms to create short videos about lottery rules, rational playing methods, and stories of responsible players. Collaborate with campus influencers while adhering to the principles of responsible gambling.

In customer support, given that college students have limited funds, financial advice should be provided to help them budget for lottery purchases. Share tips on responsible spending through online resources like blogs. Additionally, establish feedback channels such as questionnaires and online forums to collect students’ opinions on lottery products, aiming to improve these products and maintain the standards of responsible gambling.

However, the factors affecting college students’ purchasing behavior in sports lottery go far beyond that. In addition to individual psychological characteristics, external factors such as the family’s economic status, parents’ lottery purchasing habits, and social and cultural background also play an important role. Meanwhile, through tracking research, we can observe the trends of college students’ lottery purchasing behavior at different stages and the relationship between these changes and various influencing factors. In addition, the future will further enrich the consumption attitudes of different target groups toward sports lotteries, such as workers who have just entered the workforce and those who have entered the middle age stage. They can also compare the consumption trends of multiple groups to better make industrial decisions and corporate strategies.

## Data Availability

The original contributions presented in the study are included in the article/supplementary material, further inquiries can be directed to the corresponding author.

## References

[ref1] Ariyabuddhiphongs V. (2011). Lottery gambling: a review. J. Gambl. Stud. 27, 15–33. doi: 10.1007/s10899-010-9194-0, PMID: 20432057

[ref2] Beckert J. Lutter M. (2013). Why the poor play the lottery: sociological approaches to explaining class-based lottery play. Sociology 47, 1152–1170. doi: 10.1177/0038038512457854, PMID: 39917414

[ref3] Brown S. Lu Y. Ray S. Teo M. (2018). Sensation seeking and hedge funds. J. Financ. 73, 2871–2914. doi: 10.1111/jofi.12723

[ref4] Browne M. Delfabbro P. Thorne H. B. Tulloch C. Rockloff M. J. Hing N.. (2023). Unambiguous evidence that over half of gambling problems in Australia are caused by electronic gambling machines: results from a large-scale composite population study. J. Behav. Addict. 12, 182–193. doi: 10.1556/2006.2022.00083, PMID: 36729109 PMC10260219

[ref5] Chen C. Zhang Z. Long Y. (2014). Is the association between leisure-time physical activity and lottery buying habits? A cross-sectional community-based study. Med. Sci. Sports Exerc. 46:487. doi: 10.1249/01.mss.0000494927.47519.d0, PMID: 30958151

[ref6] Clor-Proell S. M. Guggenmos R. D. Rennekamp K. (2020). Mobile devices and investment news apps: the effects of information release, push notification, and the fear of missing out. Account. Rev. 95, 95–115. doi: 10.2308/accr-52625

[ref7] Cox D. F. (1967). Risk handling in consumer behavior – an intensive study of two cases. J. Mark. Res. 2, 216–228. doi: 10.1016/S1567-4223(03)00025-5

[ref8] Cui J. (2019). Research on the influence of online promotion type to perceived risk and impulse purchasing intention of consumer——Based on the moderating effects of E-commerce type and perceived express service quality Jilin University. Changchun, China: Jilin University.

[ref9] Cunningham S. M. (1967). The major dimensions of perceived risk. Cambridge, Massachusetts, United States: Boston Harvard University Press, 82–108.

[ref10] Du Z. Wang K. Li M. (2019). Promoting crowdfunding with lottery: the impact on campaign performance. Inf. Manag. 56:103159. doi: 10.1016/j.im.2019.04.002

[ref11] Dursun M. T. Argan M. Argan M. T. Dinç H. (2023). Sensation seeking and conspicuous consumption in event-based activities: the mediation role of fear of missing out (FOMO). Int J. Event Festiv. Manag. 14, 488–502. doi: 10.1108/ijefm-02-2023-0020

[ref12] Farhat R. Khan B. M. Khan A. F. (2012). Finding congruence between sensation seeking, Brand personality and purchase intention: an empirical study. Int. J. Green Comput. 3, 72–86. doi: 10.4018/jgc.2012010106

[ref13] Hedenus A. (2011). Finding prosperity as a lottery winner: presentations of self after Acquisition of Sudden Wealth. Sociology 45, 22–37. doi: 10.1177/0038038510387197, PMID: 39917414

[ref14] Kiss B. L. Deak A. Veszprémi M. D. Blénessy A. Zsido A. N. (2024). The role of excitement and enjoyment through subjective evaluation of horror film scenes. Sci. Rep. 14:2987. doi: 10.1038/s41598-024-53533-y, PMID: 38316876 PMC10844225

[ref15] Ko Y. J. Chang Y. Jang W. Sagas M. Spengler J. O. (2017). A hierarchical approach for predicting Sport consumption behavior: a personality and needs perspective. J. Sport Manag. 31, 213–228. doi: 10.1123/jsm.2015-0142

[ref9001] Li D. Zhang W. Li X. Zhen S. Wang Y. (2010). Stressful life events and problematic Internet use by adolescent females and males: A mediated moderation model. Computers in Human Behavior, 26, 1199–1207. doi: 10.1016/j.chb.2010.03.031

[ref16] Martha C. Sanchez X. Gomà-i-Freixanet M. (2009). Risk perception as a function of risk exposure amongst rock climbers. Psychol. Sport Exerc. 10, 193–200. doi: 10.1016/j.psychsport.2008.07.004

[ref17] Meng Y. Li M. He J. (2023). Sensation seeking and social network addiction among college students: a moderated mediation mode. Curr. Psychol. 43, 10979–10988. doi: 10.1007/s12144-023-05189-6

[ref18] Na L. (2021). Gambling impulsivity characteristic of moderate risk gamblers under the circumstances of sports lottery consumption. J. Tianjin Univ. Sports 36, 339–346. doi: 10.13297/j.cnki.issn1005-0000.2021.03.013

[ref19] Preacher K. J. Hayes A. F. (2004). SPSS and SAS procedures for estimating indirect effects in simple mediation models. Behav. Res. Methods Instrum. Comput. 36, 717–731. doi: 10.3758/BF03206553, PMID: 15641418

[ref20] Preacher K. J. Rucker D. D. Hayes A. F. (2007). Addressing moderated mediation hypotheses: theory, methods, and prescriptions. Multivar. Behav. Res. 42, 185–227. doi: 10.1080/00273170701341316 26821081

[ref21] Przybylski A. K. Murayama K. DeHaan C. R. Gladwell V. (2013). Motivational, emotional, and behavioral correlates of fear of missing out. Comput. Hum. Behav. 29, 1841–1848. doi: 10.1016/j.chb.2013.02.014, PMID: 39927108

[ref22] Sanmartín F. J. Velasco J. Gálvez-Lara M. Cuadrado F. Moriana J. A. (2023). Risk factors associated with gambling on loot boxes. Psicothema 35, 397–405. doi: 10.7334/psicothema2022.484, PMID: 37882424

[ref23] Shapiro S. L. Reams L. So K. K. F. (2019). Is it worth the price? The role of perceived financial risk, identification, and perceived value in purchasing pay-per-view broadcasts of combat sports. Sport Manag Rev 22, 235–246. doi: 10.1016/j.smr.2018.03.002

[ref24] Shepperd J. A. Forsyth R. B. (2023). How Does Religion Deter Adolescent Risk Behavior? Curr. Dir. Psychol. Sci. 32, 337–342. doi: 10.1177/09637214231164404

[ref25] Shin S. H. Montalto C. P. (2015). The role of impulsivity, cognitive bias, and reasoned action in understanding college student gambling. J. Youth Stud. 18, 376–395. doi: 10.1080/13676261.2014.963537, PMID: 39918549

[ref26] Sport G. A. O. (2024a). “Public welfare sports lottery happy playground” Sets Sail in Henan. Available at: https://www.sport.gov.cn/n20001280/n20067608/n20067637/c27761456/content.html (Accessed August 31, 2024).

[ref27] Sport G. A. O. (2024b). Sports lottery report card for 2023: Responsible lottery construction reaches New Heights. Available at: https://www.sport.gov.cn/n20001280/n20067608/n20067637/c27761456/content.html (Accessed August 31, 2024).

[ref28] Sport G. A. O. (2024c). New practice of promoting high-quality development of sports lottery with Chinese modernization. Available at: https://www.sport.gov.cn/n315/n20067006/c27670237/content.html (Accessed August 31, 2024).

[ref29] Steinberg L. Albert D. Cauffman E. Banich M. Graham S. Woolard J. (2008). Age differences in sensation seeking and impulsivity as indexed by behavior and self-report: evidence for a dual systems model developmental psychology. Dev. Psychol. 44, 1764–1778. doi: 10.1037/a0012955, PMID: 18999337

[ref30] Tamon H. Itahashi T. Yamaguchi S. Tachibana Y. Fujino J. Igarashi M.. (2022). Autistic children and adolescents with frequent restricted interest and repetitive behavior showed more difficulty in social cognition during mask-wearing during the COVID-19 pandemic. BMC Psychiatry 22:608. doi: 10.1186/s12888-022-04249-8, PMID: 36104779 PMC9471034

[ref31] Wagner P. Duan Y. P. Zhang R. Wulff H. Brehm W. (2020). Association of psychosocial and perceived environmental factors with park-based physical activity among elderly in two cities in China and Germany. BMC Public Health 20:55. doi: 10.1186/s12889-019-8140-z, PMID: 31937268 PMC6961356

[ref32] Wegmann E. Oberst U. Stodt B. Brand M. (2017). Online-specific fear of missing out and internet-use expectancies contribute to symptoms of internet-communication disorder. Addict. Behav. Rep. 5, 33–42. doi: 10.1016/j.abrep.2017.04.001, PMID: 29450225 PMC5800583

[ref33] Wu Y. Li H. Li W. (2020). Applying a psychotherapeutic theory to the modeling of affective intelligent agents. IEEE Trans. Cogn. Dev. Syst. 12, 285–299. doi: 10.1109/tcds.2019.2911643, PMID: 39573497

[ref34] Xie X. Wang Y. Wang P. Zhao F. Le L. (2018). Basic psychological needs satisfaction and fear of missing out: friend support moderated the mediating effect of individual relative deprivation. Psychiatry Res. 268, 223–228. doi: 10.1016/j.psychres.2018.07.025, PMID: 30064069

[ref35] Xu J. Li H. Wu Y. Dong C. Zhang Z. (2023). Compiling and evaluating the scale of gambling behavior of numbers and lotto lottery: an experimental analysis based on China sports lottery consumers. J. Shandong Sport Univ. 39, 44–53+74. doi: 10.14104/j.cnki.1006-2076.2023.02.006

[ref36] Zhou H. Wu A. M. S. (2024). The protective effects of cognitive empathy and emotional empathy on gambling disorder are mediated by risk aversion and responsible gambling attitude. BMC Psychiatry 24:63. doi: 10.1186/s12888-024-05509-5, PMID: 38254048 PMC10804480

[ref37] Zuckerman M. (1990). The psychophysiology of sensation seeking. J. Pers. 58, 313–345. doi: 10.1111/j.1467-6494.1990.tb00918.x 2198341

[ref38] Zuckerman M. (1996). The psychobiological model for impulsive unsocialized sensation seeking: a comparative approach. Neuropsychobiology 34, 125–129. doi: 10.1159/000119303, PMID: 8916069

[ref39] Zuckerman M. (2014). Sensation seeking (psychology revivals). London: Psychology Press.

